# Clinical Blindness to Eating Disorders in Older Adults: A Qualitative Study of Primary Care Nurses’ Perspectives

**DOI:** 10.3390/healthcare14162586

**Published:** 2026-08-17

**Authors:** Adriana V. Muñoz-Ortega, Lorenzo Mariano Juárez, Mikaela Willmer, Paula Conroy, Maria Engström, David Conde Caballero

**Affiliations:** 1 Department of Computer and Telematics Engineering, School of Engineering, University of Extremadura, 10003 Cáceres, Spain; 2 Interdisciplinary Group for Studies in Society, Culture and Health, University of Extremadura, 10003 Cáceres, Spain; lorenmariano@unex.es (L.M.J.); dcondecab@unex.es (D.C.C.); 3 Department of Psychology and Anthropology, Faculty of Teacher Training, University of Extremadura, 10003 Cáceres, Spain; 4 Department of Caring Science, Faculty of Health and Occupational Studies, University of Gävle, 801 76 Gävle, Sweden; mikaela.willmer@hig.se (M.W.); maria.engstrom@hig.se (M.E.); 5 Department of Sport, Exercise and Nutrition, Atlantic Technological University, H91 T8NW Galway, Ireland; paula.conroy@atu.ie; 6 Department of Nursing, Faculty of Medicine and Health Sciences, University of Extremadura, 10003 Cáceres, Spain

**Keywords:** eating disorders, older adults, primary care, nursing, qualitative research, ageism

## Abstract

**Background/Objectives**: Eating disorders (EDs) have historically been associated with young women, and their presence in older adults remains poorly recognized despite evidence that they can persist, recur, or emerge in later life. Primary care nurses are strategically positioned for community-based detection, yet their perspective has been scarcely explored. This study aimed to examine the experiences, perceptions, and described practices of primary care nurses regarding EDs and disordered eating in older adults, identifying patterns, barriers to detection, and training needs. **Methods**: A qualitative study with a descriptive design was conducted in three Primary Health Care Centers from different health areas of Spain. Thirty-two registered practicing nurses participated in three focus groups, guided by a clinical vignette and a semi-structured interview guide. Data were analyzed following Braun and Clarke’s reflexive thematic analysis, supported by ATLAS.ti software (version 24.2, 2024). **Results**: EDs in older adults frequently remained invisible to professionals, sustained by three interrelated mechanisms: the cultural hegemony of the biomedical paradigm, which reframes symptoms as organic or cognitive; a selective historicization that attributes altered eating to the postwar “hunger years”; and an “ageism of care” that normalizes warning signs as expected features of aging. Notably, when the diagnostic possibility was explicitly named, participants retrospectively reinterpreted cases they had previously explained in other terms. **Conclusions**: This invisibility stems not from an absence of clinical signs, but from professional, cultural, and institutional interpretive frameworks. Findings underscore the need for specific training, adapted screening tools, and a critical perspective on ageism in nursing practice.

## 1. Introduction

Eating disorders (EDs) are persistent disturbances of eating-related behaviors that cause significant impairment in the physical and mental health of those affected. Both this definition and its diagnostic criteria are set out in the fifth edition of the Diagnostic and Statistical Manual of Mental Disorders (DSM-5), which encompasses entities such as anorexia nervosa, bulimia nervosa, binge-eating disorder, and other specified and unspecified feeding and eating disorders [1]. Over time, clinical and scientific interest has progressively expanded beyond these diagnostic categories to incorporate the concept of disordered eating—a construct that captures a broad spectrum of dysfunctional eating behaviors and problematic attitudes toward food, body weight, and physical appearance [2]. This continuum includes practices such as persistent restriction of intake, often referred to as “chronic dieting” [3], compulsive calorie monitoring, rigid avoidance of specific foods, and excessive preoccupation with weight and body appearance [4]. These behaviors are not isolated: population-based studies in adults have identified them in 10% of the sample and have further documented that they can precede or coexist with a fully diagnosable ED [5].

A notable feature of EDs is that they have historically been tied to a stereotyped image: that of young, White, underweight women [6,7]. This representation has shaped scientific output, the development of clinical guidelines, and the training of health professionals, channeling attention almost exclusively toward the earliest stages of the life course. The very design of diagnostic systems has helped entrench this age-related bias; in fact, as early as 1980, the DSM-III restricted the diagnosis of anorexia nervosa to individuals under 30 years of age, consolidating an understanding of EDs closely tied to youth [8].

In contrast, evidence shows that EDs can persist, recur, or even emerge for the first time in old age. Pioneering work and its recent updates have documented new diagnoses after age 65, as well as periods of particular vulnerability associated with the menopausal transition [9,10]. A review published in 2019 reported prevalence rates ranging from 1.5% to 6% in adults over 65 [11], whereas subsequent estimates placed this figure between 1.8% and 3.8% for women over 60 [10]. Although the available estimates are heterogeneous, there is consensus that EDs in later life represent a clinically relevant problem, associated with significant morbidity and potentially elevated mortality [12].

However, this clinical weight contrasts with the limited attention that this age group has received in the scientific literature. A recent systematic review on the treatment of EDs in older adults identified only observational studies and case series, highlighting the absence of clinical trials, and consequently the lack of clinical practice guidelines tailored to this population [12]. This invisibility is particularly relevant in the primary care setting, the main level of care for the follow-up of older adults and for the early detection of potential nutritional problems. Evidence has consistently shown the association between inadequate food intake, malnutrition, frailty, and sarcopenia—geriatric syndromes linked to a greater risk of dependence, institutionalization, and mortality [13,14]—which underscores the importance of their early diagnosis. Against this backdrop, EDs and other disordered eating behaviors could constitute an underexplored factor in old age, fostering patterns of restriction, avoidance, or insufficient intake. Their identification therefore takes on growing relevance in a context of progressive population aging.

Of all the health professionals in this care setting, nursing staff are strategically positioned for community-based detection owing to their direct contact with older adults. However, this position is not always matched by specific screening tools or by training tailored to the identification of EDs in later life, which makes it difficult to distinguish between the physiological changes of aging, underlying organic disease, and possible disorders [15,16]. The absence of validated instruments and nursing-specific tools for this age group may contribute to the underdetection already noted and to the normalization of signs that could carry clinical relevance [17].

Despite the importance of this issue, the perspective of nursing staff on this problem has been scarcely explored. The existing literature has focused mainly on malnutrition, loss of appetite, or general nutritional interventions at this level of care [18], whereas the processes of clinical interpretation and day-to-day management of these behaviors have received limited attention. Recovering this professional experience is essential, as it can provide relevant evidence to inform the design of screening and intervention strategies in primary care and help improve the detection of a problem with potential clinical impact.

Within this context, the present study aims to explore the experiences, ideologies, perceptions, and described practices of primary care nursing staff in relation to EDs and disordered eating behaviors in older adults, with the goal of identifying common patterns, barriers to detection, and training needs in this care setting, as well as broadening the focus on problems that may have an impact on frailty and functional decline.

## 2. Materials and Methods

A qualitative study with a descriptive design was conducted, based on a focus group methodology and using an inductive analysis of the data.

### 2.1. Setting and Participants

Fieldwork was carried out in three Primary Health Care Centers belonging to different health areas of Spain. Each center constitutes a Basic Health Zone, the territorial unit of reference for the organization and delivery of primary care within the Spanish National Health System, with a registered population of approximately 15,000 to 20,000 inhabitants each. The centers were purposively selected in order to incorporate organizational and sociodemographic diversity while maintaining a comparable care structure.

Participants were recruited through convenience sampling, with the collaboration of the management teams of the participating centers. All participants were registered practicing nurses. The sample comprised 32 professionals (Table 1): 20 women and 12 men. As an inclusion criterion, a minimum of six months of experience in the primary care and regular care of older adults was required. This criterion ensured sufficient familiarity with day-to-day clinical practice and with the challenges of caring for the older adult population in the community setting. Professionals with less than six months of clinical experience or without regular contact with older adults in their clinical practice were excluded.

### 2.2. Data Collection and Procedure

Data collection was carried out between January and May 2026. The focus group technique was selected for its capacity to promote interaction among participants and to facilitate the exploration of shared experiences, perceptions, and practices regarding complex phenomena, allowing for the identification of both consensus and divergence within the same professional context [19]. Three focus groups were conducted, one at each participating Primary Health Care Center. The groups comprised 12, 12, and 8 professionals, respectively. The size of the groups was determined by the number of professionals available and willing to participate at each center. Although focus groups are frequently recommended to comprise six to eight participants, larger groups are considered appropriate when participants share a common professional frame of reference and the discussion focuses on their expertise rather than on sensitive personal experiences [19].

Prior to the start of fieldwork, an interdisciplinary international research team (nurses, anthropologists, and dietitians) with extensive expertise in EDs developed a clinical vignette designed to stimulate discussion and provide a common frame of reference. The vignette described the case of an older adult under care presenting with weight loss, persistent alterations in eating patterns, and food avoidance behaviors potentially consistent with an ED or with disordered eating. Based on this scenario, a semi-structured interview guide was developed, comprising open-ended questions aimed at exploring the experiences, perceptions, and practices of the professionals regarding the identification and management of these situations. The guide was adapted to the primary care setting, paralleling the procedure previously used by the team in residential care settings [17] (Table 2).

### 2.3. Research Team and Roles

The research team was multidisciplinary, comprising professionals from nursing, nutrition, and the social sciences with experience in qualitative research and in the field of nutrition and aging. This composition made it possible to approach the phenomenon under study from complementary clinical, nutritional, and sociocultural perspectives, fostering a broader understanding of the factors involved in the identification and management of these disorders.

The interview guide was designed collaboratively by the research team, drawing on the available scientific literature and on the members’ clinical experience. Prior to its use, the clinical vignette and the questions were reviewed by professionals with experience in the care of older adults, in order to ensure their clarity, clinical relevance, and suitability for the primary care setting.

### 2.4. Conduct of the Focus Groups

The sessions were scheduled at times agreed upon with the participating centers so as not to interfere with clinical activity. The center coordinators provided initial information about the study to potential participants, whose participation was entirely voluntary.

The sessions began with the reading of the clinical vignette and a brief period of individual reflection. The moderator then introduced the questions in order to explore in greater depth the perceptions, experiences, and practices of the professionals. Two members of the research team took part in each focus group. The moderator led the discussion, encouraged balanced participation, and probed emerging themes. The second researcher acted as an observer, recording contextual aspects, group dynamics, nonverbal elements, and instances of consensus or disagreement relevant to data interpretation.

### 2.5. Data Analysis

The recordings were transcribed verbatim and underwent an anonymization process through the removal of any potentially identifying information. The data were stored in a secure, password-protected repository accessible only to the research team.

The analysis followed the reflexive thematic analysis described by Braun & Clarke [20,21], an approach well-suited to exploring how professionals experience, interpret, and account for the identification and management of these situations. Each transcript was first read in full several times to obtain a sense of the whole. Initial codes were then generated systematically across the entire dataset, capturing segments relevant to the study objectives. Related codes were subsequently collated to construct candidate themes, which were reviewed against both the coded extracts and the dataset, and then refined, defined, and named. Consistent with the reflexive orientation of the method, coding was understood as an interpretative and situated process rather than as the neutral extraction of pre-existing content, and the disciplinary backgrounds of the research team (nursing, anthropology, and dietetics) were treated as an analytic resource. Data organization and coding were supported by ATLAS.ti software (version 24.2, 2024).

Each participant was assigned a code indicating the focus group and the individual within that group (e.g., Focus Group 1-P3), with participant numbering corresponding to the order presented in Table 1. These codes are used throughout Section 3 to attribute each quotation to a specific participant. Individual speaker codes were not linked to any identifying information, in order to preserve the confidentiality of participants belonging to relatively small professional teams.

Data collection and analysis were conducted in Spanish. The verbatim quotations presented in the results were translated into English by the research team, with attention to preserving the meaning and tone of the original expressions.

To strengthen credibility and analytical consistency, several researchers independently took part in the review of emerging categories. Discrepancies were discussed until consensus was reached, ensuring that the interpretations remained grounded in the data and adequately reflected the experiences expressed by the participants.

### 2.6. Ethical Considerations

All participants received verbal and written information about the study’s objectives, the voluntary nature of participation, the confidentiality of the data, and their right to withdraw at any time without the need for justification. They subsequently provided written informed consent.

Confidentiality was ensured through the anonymization of the transcripts and the exclusive use of coded data during the analytical process. The study was approved by the Bioethics Committee of the University of Extremadura (114/2026) for the EDOP-26 Project and was conducted in accordance with the ethical principles set out in the Declaration of Helsinki and with current data protection legislation. This approval constitutes a continuation of the same research protocol, initially approved by the same Committee in 2024 (No. 161/2024); the 2026 approval corresponds to the ongoing development of that protocol rather than to a separate study.

### 2.7. Reflexivity

The research team incorporated reflexivity as a transversal component of the research process, recognizing that the professional trajectories, prior experiences, and disciplinary frameworks of the researchers may shape the production and interpretation of knowledge. The multidisciplinary nature of the team fostered critical discussion of emerging interpretations and the consideration of complementary perspectives throughout the analytical process. It was also acknowledged that the researchers’ position as health professionals and academics could influence focus group dynamics and the discourses produced by the participants. To minimize this effect, moderation was oriented toward fostering an open, respectful, and non-hierarchical environment that facilitated the expression of diverse views. Throughout the analysis, ongoing reflexive vigilance was maintained in order to avoid the imposition of preconceived categories and to ensure that interpretations remained grounded in the participants’ accounts and experiences.

## 3. Results

The analysis of the focus groups revealed a complex framework in which EDs in older adults often remain invisible to nursing professionals, largely due to cultural and professional barriers. This invisibility does not stem from the absence of symptoms—which professionals recognize when invited to reflect on them—but from specific sociocultural constructions that shape their clinical interpretation. The analysis identified at least three interrelated mechanisms that structure and sustain this invisibility in the Spanish context.

### 3.1. Category 1: Cultural Hegemony of the Biomedical Paradigm

Nursing professionals tend to systematically attribute the symptoms associated with EDs in older adults to organic pathologies, medical comorbidities, or cognitive impairment. This tendency reflects the hegemony of the biomedical paradigm as the dominant framework of clinical interpretation, one that privileges organic explanations over approaches centered on eating behavior or mental health.

*“I think it’s more because of the illness she has, not because of … ‘I’m fatter, I’m thinner’, I think… Not because of her appearance. I don’t think it’s about her appearance either …”*

(Focus Group 1-P3)

*“Being a smoker too, I think… I mean, I’m not a smoker, but I imagine food doesn’t taste the same to her and … Or the medication they’re on. A lot of medications affect appetite, make you not feel like eating, and well, and COPD—the COPD picture often goes along with cachexia. In the end people are generally very thin, and on top of that, a smoker—well, you can just imagine. And if she has dementia, it could also be that… she even forgets to eat. I mean, that could happen, that she forgets…”*

(Focus Group 3-P5)

*“Exactly. Besides, with so many comorbidities, you always find a medical explanation. There’s always diabetes, depression, cognitive impairment… So many factors are involved that it’s easy to attribute it to that and not think of any other kind of disorder.”*

(Focus Group 2-P7)

Across the groups, a recurrent movement emerged. Participants who had initially denied the presence of EDs in older adults, or explained the observed behaviors through other causes, went on to describe eating patterns that closely resembled recognized features of these disorders, yet without labeling them as such. This shift became particularly apparent when the diagnostic theme was explicitly named within the session, prompting several participants to revisit specific cases they had previously framed in other terms. This is illustrated by the following account:

*“Now that you mention it, there’s a woman where you have to be constantly telling her eat, eat … and she doesn’t even make the attempt to eat, I mean, she doesn’t want to eat. I’ll eat later, I’ll eat later, I’ll eat later, but there’s the plate, and unless you force her—not force her, but unless you help her with the act of eating … she doesn’t eat. The idea of sitting down to eat doesn’t appeal to her, this could be what you’re talking about …”*

(Focus Group 1-P12)

The same reframing appeared in another group, where naming the diagnostic possibility led a participant to reinterpret a case in light of a long-standing history:

*“Well, now that I’m thinking about this, I remember the case of a woman who … I think she had been anorexic her whole life. And she came here and had the same thing … I think that woman, because her daughter mentioned it, had had that disorder from way back.”*

(Focus Group 2-P1)

In both instances, behaviors that had not previously been read as signs of an ED were reinterpreted once the possibility was made available within the group.

Beyond this individual interpretive level, the hegemony of the biomedical paradigm also manifests itself in structural dimensions. In particular, it operates as an institutionalized framework that shapes both professional training and the design of clinical tools, limiting the capacity for the systematic detection of EDs in later life. The absence of screening protocols specific to the older population and the training gaps explicitly acknowledged by the professionals contribute to consolidating this divide between clinical knowledge and diagnostic recognition.

*“’Do you have any kind of training on eating disorders in older people?’ No. Specifically on anorexia and bulimia in older adults, we don’t have anything …”*

(Focus Group 3-P8)

Taken together, these findings suggest that the invisibility of EDs in old age cannot be attributed solely to a low prevalence or to the absence of clinically relevant cases, but may also be attributed to a sociocultural and institutional process of restricted clinical framing, in which the biomedical paradigm operates simultaneously as an interpretive filter and as an organizing structure of healthcare knowledge.

### 3.2. Category 2: Selective Historicization. The Weight of the “Hunger Years”

A particularity of the Spanish case is that professionals draw on historical narratives that are widespread in the country’s collective imaginary to account for all kinds of anomalous eating behaviors in old age, establishing a direct—and at times oversimplified—causal link between past collective experiences and current conduct. This form of selective historicization functions as an interpretive mechanism that, while providing narrative coherence, can also operate as a device of simplification, reducing complex life trajectories to homogeneous generational attributes and potentially displacing other clinically relevant hypotheses. In the Spanish case, this explanatory framework systematically points to experiences of food scarcity during the postwar period and the so-called “hunger years” (the 1940s), shaping an interpretive repertoire that is widely shared in professional discourse:

*“It happens a lot because of the Civil War, a lot … well, there aren’t that many left now, but … having gone hungry, ‘I have to eat and make the most of what there is now,’ and they eat more than they need …”*

(Focus Group 1-P1)

When accounting for the eating behaviors of older adults, participants frequently drew on the collective, biographical and historical experience of deprivation during the Spanish postwar period, called the “hunger years”, as an explanation for reduced or altered eating.

*“The generation that lived through the Civil War has that mindset of making the most of everything. They don’t throw anything away and they eat as much as they can ‘just in case.’ These are people who went through a lot of need, so now it’s very hard for them to leave food on the plate or throw anything out. There’s always that ‘this gets saved,’ ‘this gets used,’ or ‘this in case there’s nothing tomorrow.’ In the end it’s like a way of life they’ve carried with them since they were small, because they lived through very hard times, of scarcity, and that really shows in how they relate to food. It’s not so much that there’s a problem, but that they come from that experience.”*

(Focus Group 3-P2)

### 3.3. Category 3: Cultural Construction of Old Age. The Ageism of Care

The invisibility of EDs cannot be explained solely by the factors previously described; it is also shaped by a shared sociocultural construction of old age that conditions perception from the outset, directly affecting the detection of this and other problems. In this sense, what might be termed the “ageism of care” operates as an interpretive framework that determines clinical perception and contributes to the invisibility of EDs. Within this framework, potentially problematic eating behaviors tend to be reinterpreted as normative expressions of aging. Behaviors such as the restriction of food intake, avoidance of certain foods, or a conflictive relationship with food are frequently described as “quirks” or “things that come with age”, rather than as possible indicators of EDs. In this context, age-related prejudices delimit the horizon of what is clinically thinkable, ruling out the possibility that older adults may experience concerns related to body image or weight control. As one participant noted:

*“The elderly people we care for don’t worry much about being fat or thin; older people aren’t seen that way. Maybe I always think it’s something else, and maybe … I don’t know…”*

(Focus Group 1-P4)

This interpretive framework would contribute to the construction of a homogeneous image of old age, in which older adults are conceived as a uniform group with shared needs and characteristics. This homogenization renders invisible the diversity of life trajectories and subjective experiences, with direct implications for care practice, as it reduces sensitivity to mental health problems that may potentially be present. In this sense, ageism does not operate solely as an individual bias, but as a new organizing logic of care that runs across different levels of healthcare. This logic is reflected both in the structuring of care responses and in the interpretation of psychological distress in the older population:

*“We’ve had cases of users who present episodes of compulsive eating and then report stomach discomfort and even end up vomiting. But we have always thought it was just things that come with age, or that it had to do with other pathologies …”*

(Focus Group 2-P9)

*“I think that when these things happen it’s more from boredom typical of old age or from anxiety, more than from anything else …”*

(Focus Group 2-P11)

## 4. Discussion

The results of this study reveal that EDs in older adults remain systematically invisible, sustained by at least three interconnected mechanisms that operate simultaneously in the Spanish context. The analysis shows how professional, cultural, and structural factors act as epistemic barriers that maintain a “clinical blindness” toward a serious condition with potential impact on frailty. The interpretive dimension was made particularly visible within the focus groups themselves: when the diagnostic theme was explicitly named, several participants retrospectively reinterpreted cases they had previously explained in other terms. This finding is particularly relevant in a context marked by progressive demographic aging, in which approximately one-third of the population will be over 65 years of age by 2064, and the dependency ratio will nearly double the current levels [22].

### 4.1. Biomedical Reductionism as a Cultural Filter

The first of the identified mechanisms relates to the hegemony of the biomedical paradigm in clinical practice. Our results show that health professionals tend to systematically attribute behaviors consistent with EDs to physical pathologies or cognitive impairment, thereby displacing their psychological and social dimensions. This process aligns with what O’Callaghan [23] terms the “cultural filter” of biomedical reductionism, through which symptoms are reframed within alternative medical explanations, reproducing in the field of EDs the pattern already described by Engel [24] in his critique of the biomedical model. This is neither a new nor an exclusive trend. Medical anthropology [25], clinical psychology [26], and medicine itself have repeatedly highlighted the impact of “biological reductionism” [27], which privileges organic signs and tends to render invisible the social and psychological determinants essential to diagnosis in mental health, including EDs [28].

The lack of specific training on EDs in old age has also been documented in the previous literature [12]. However, our findings allow this point to be nuanced: such a gap cannot be interpreted solely as a technical or educational limitation amenable to isolated remediation, but rather as the expression of a broader epistemological framework that has placed these problems at the margins of clinical legitimacy, hindering their recognition within professional practice.

This entire web generates a feedback loop: limited training and the scarce availability of diagnostic tools contribute to the clinical invisibility of EDs, which in turn reduces the demand for new tools and slows their development. Systematic reviews have noted that the lack of systematic screening and insufficient professional training constitute central barriers to the detection and management of these disorders [28]. Our results confirm that these are not isolated deficits but structural exclusions that have historically placed EDs outside the recognized field of intervention in old age [12]. This pattern is not exclusive to EDs; it has also been observed in other conditions such as fibromyalgia and chronic fatigue syndrome, underscoring the persistence of structural biases in healthcare that limit the visibility and clinical management of complex, multifactorial problems [29].

### 4.2. Cultural-Historical Determinism as a Mechanism of Normalization

The second barrier identified reveals a mechanism of particular importance in the Spanish context: the tendency to interpret certain eating behaviors of older adults through biographical and historical narratives linked to experiences of deprivation during the postwar period. In the discourses analyzed, references to the so-called “hunger years” [30] recurrently emerged as an explanatory resource for understanding food-related behaviors. Although these interpretations refer to real and socially significant historical experiences, our findings suggest that their continued use may operate as a form of cultural determinism that shapes clinical assessment.

The literature has extensively documented the persistence of the “traumatic memory of hunger” and its influence on food practices long after the events that gave rise to it [31,32]. This phenomenon has been described in contexts beyond the Spanish one, such as the Great Irish Famine [33,34], the Chinese famine of 1959–1961 [35], and the Ukrainian Holodomor [36], where experiences of scarcity left a lasting imprint on individual and collective relationships with food. However, the contribution of our study does not lie in confirming the persistence of these memories, but in showing how they are incorporated into everyday clinical reasoning as explanatory categories for certain eating behaviors observed in older adults. From this perspective, the issue is not the historical validity of these narratives, but their interpretive consequences. When loss of appetite, restrictive behaviors, or food avoidance are primarily explained on the basis of past experiences of scarcity, the risk increases that other potentially relevant factors—including EDs—will be sidelined. Rather than fostering a comprehensive understanding of the clinical situation, these explanations may contribute to normalizing behaviors that in other age groups would be considered indicative of pathology.

This finding broadens our understanding of the mechanisms that hinder the recognition of EDs in old age. While the previous literature has emphasized the role of cultural factors in the shaping and expression of these disorders, particularly in younger populations [37], our results suggest that in older adults, such factors may operate differently: not as elements that facilitate understanding of the problem, but as interpretive devices that contribute to its deproblematization. This normalizing function of culture has received scant attention in the literature and highlights the need to develop clinical approaches capable of integrating the biographical and historical trajectories of older adults without this leading to minimizing or dismissing the presence of eating-related problems amenable to assessment and intervention.

### 4.3. The “Ageism of Care” as a Cross-Cutting Mechanism of Invisibility

Our results indicate that health professionals tend to minimize certain warning signs in older adults, interpreting them as expected manifestations of aging. We propose conceptualizing this phenomenon through the notion of “ageism of care”: a mechanism of invisibility affecting older adults that transcends individual biases to take on a structural and epistemic dimension [38]. We use this notion to designate a construct more specific than the broader concept of ageism in healthcare described in the literature [38]. Whereas the latter refers to age-based bias across healthcare systems and institutions in general, our term seeks to capture the way these assumptions operate within the particular domain of nursing care, where the normalization of warning signs is embedded in everyday caring practices and in the relational construction of what is considered “normal” in old age. Naming this specificity is intended to render visible a phenomenon that a more general framing may leave unexamined.

However, this minimization of symptoms is not confined to the realm of eating behaviors. The available evidence shows that similar processes affect multiple clinical conditions and symptoms in old age [39]. In care settings, it is common for manifestations such as pain, fatigue, or functional decline to be interpreted as inevitable consequences of aging [40]. This logic is present not only in professional assessments, but also in the accounts of older adults themselves, who may come to internalize these explanations and normalize potentially pathological experiences. In this way, age-based assumptions mutually reinforce one another in the interaction between patients, professionals, and institutions [41].

These mechanisms have been extensively documented in the scientific literature and are embedded in broader social contexts in which age-based prejudices are highly normalized [42]. According to estimates from the World Health Organization, approximately half of the world’s population holds moderate or high ageist attitudes at the general-population level [43]; evidence focused specifically on the nursing profession has likewise documented the presence of ageist attitudes toward older adults, which appear to have grown more negative over the last decade [44,45]. These attitudes may influence clinical decision-making and contribute to differences in the quality of care provided to older adults compared with younger patients with equivalent medical conditions. From a structural perspective, ageism of care is also reproduced through the very mechanisms of the production and validation of clinical knowledge. The development of diagnostic and screening instruments based predominantly on younger populations [46] is a paradigmatic example. As a consequence, a structural legitimization of professional neglect is generated, contributing to the perpetuation of existing diagnostic and therapeutic gaps.

### 4.4. Implications for Clinical Practice and Public Health

The findings of this study have direct implications for clinical practice in a context of progressive population aging, in which malnutrition, frailty, and dependency constitute priority public health problems [47]. In this scenario, the assessment of nutritional status in older adults takes on a central role, not only as a health indicator, but as a key determinant of the loss of functionality and autonomy. However, our results indicate that EDs and other disordered eating behaviors remain underdetected in old age, despite their possible contribution to these processes.

These findings suggest the need to broaden the clinical perspective beyond traditional nutritional markers, systematically incorporating behavioral and psychosocial dimensions of eating, in line with what has been indicated by the World Health Organization [48]. Early detection does not depend solely on clinical criteria, but also on the interpretive frameworks through which professionals understand eating in old age, in which biomedical reductionism, ageism, and certain sociocultural narratives can contribute to the normalization of warning signs and to their underreporting.

Recognizing EDs in later life may also require clinicians to reconsider health problems that in older adults are often attributed to aging or to isolated organic causes, but which can also signal an underlying disturbance of eating. Manifestations such as falls, syncope, cardiac arrhythmias (including QT prolongation), postural hypotension, constipation and other gastrointestinal complaints, electrolyte disturbances, dental or denture problems, and malnutrition may, in some cases, reflect the physiological consequences of restrictive eating, purging, or undernutrition [49]. Their systematic assessment and re-assessment could contribute to earlier identification. In particular, nursing professionals need to distinguish conditions that are common in aging, such as dysphagia and other swallowing difficulties [50], from the less frequently considered possibility of an ED, bearing in mind that swallowing difficulties are also common in EDs and may be both a consequence and a contributing factor of disordered eating.

Consequently, it is necessary to integrate nutritional assessment with the identification of anomalous eating behaviors within teams caring for older adults, particularly in primary care and community nursing. The need for specific training on EDs in old age and for the development of screening tools adapted to this age group is likewise reinforced.

## 5. Conclusions

This study shows that eating disorders in older adults constitute a clinically relevant yet systematically underdetected phenomenon in primary care. This invisibility does not stem from the absence of clinical signs, but from the interplay of professional, cultural, and institutional interpretive frameworks that condition their recognition.

In particular, biomedical reductionism, cultural-historical determinism, and the ageism of care operate as interrelated mechanisms that contribute to normalizing or reinterpreting potentially pathological eating behaviors within old age. Taken together, these processes shape a “clinical blindness” that limits the early identification of EDs, and with it, the possibility of intervening on their nutritional, functional, and psychosocial consequences.

In a context of progressive population aging, these findings underscore the need to rethink the frameworks of nutritional assessment in older adults, systematically integrating the behavioral and psychosocial dimensions of eating. They likewise reinforce the importance of specific training for nursing professionals, the development of screening tools adapted to this age group, and the incorporation of a critical perspective on ageism into clinical practice.

## 6. Limitations

This study presents several limitations that should be considered. First, although the qualitative design allows for an in-depth understanding of the phenomena studied, the number of focus groups and their restriction to three Primary Health Care Centers limits the transferability of the findings.

Second, the sample consisted exclusively of primary care nursing professionals, which provides a specific and valuable perspective but excludes other relevant viewpoints, such as those of medicine, clinical nutrition, or social work, as well as the direct experience of older adults themselves, whose inclusion could significantly enrich the understanding of the phenomenon. Relatedly, participants were enrolled through convenience sampling, which may introduce a risk of selection bias, as professionals with a particular interest in the topic may have been more inclined to take part. Future research could adopt purposive sampling strategies with maximum-variation criteria or probabilistic approaches to broaden the range of perspectives represented.

Third, the analysis did not unearth accounts in which EDs were confidently recognized or in which the cultural explanations invoked by participants were explicitly challenged. This convergence may reflect the composition of the sample, the recruitment strategy, or the primary care context, and larger, more diverse samples including specialized care settings would help establish whether counter-narratives emerge.

Fourth, the use of clinical vignettes may have influenced the activation of certain interpretive frameworks, fostering guided reflection on the phenomenon under study. Although this strategy facilitates the discussion of complex cases, it may also condition the spontaneity of some accounts compared with unstructured clinical practice.

Fifth, although the study incorporated organizational diversity across centers, it did not explore in depth possible cultural or regional variations within the Spanish context, which could be relevant for a more precise understanding of how the identified interpretive frameworks are constructed and reproduced.

Finally, the practical recommendations derived from this study remain necessarily general. Translating them into specific screening instruments or training models exceeds the scope of a qualitative design of this nature. Ongoing research by our team, drawing on larger samples and complementary methodologies, aims to refine these questions and move toward more concrete, empirically grounded recommendations.

## Figures and Tables

**Table 1:** Sociodemographic and professional characteristics of participants by focus group (N = 32).

Focus Group	Centre	Code	Gender	Age	Years of Experience	Job Position
Focus Group 1	Urban	P1	Female	34	5	Nurse
		P2	Female	23	1	Nurse
		P3	Female	34	3	Nurse
		P4	Female	33	3	Nurse
		P5	Male	28	2	Nurse
		P6	Female	46	10	Nurse
		P7	Female	51	15	Nurse
		P8	Male	45	5	Nurse
		P9	Male	36	7	Nurse
		P10	Male	32	9	Nurse
		P11	Male	44	10	Nurse
		P12	Female	27	4	Nurse
Focus Group 2	Urban	P1	Female	25	1	Nurse
		P2	Female	28	1	Nurse
		P3	Male	32	3	Nurse
		P4	Female	34	5	Nurse
		P5	Male	24	1	Nurse
		P6	Male	31	4	Nurse
		P7	Female	32	5	Nurse
		P8	Female	30	5	Nurse
		P9	Male	32	4	Nurse
		P10	Female	29	2	Nurse
		P11	Female	30	2	Nurse
		P12	Male	34	7	Nurse
Focus Group 3	Rural	P1	Female	24	1	Nurse
		P2	Female	36	5	Nurse
		P3	Male	55	20	Nurse
		P4	Female	46	14	Nurse
		P5	Male	36	8	Nurse
		P6	Female	34	7	Nurse
		P7	Female	32	5	Nurse
		P8	Female	58	24	Nurse

**Table 2:** Semi-structured interview guide used to facilitate focus group discussions.

Sequence	Key Questions
Opening	What are your initial thoughts when reading this case of an older person attending primary care?
Experience Recognition	Can you describe any similar situations you have encountered in your clinical practice with older adults in primary care? What happened, and how did you manage the situation?
Professional Discourse	How are food, eating behaviors, body weight, and nutritional concerns usually discussed among older adults and healthcare professionals in primary care?
Manifestation Understanding	How do you think disordered eating might manifest itself in an older person?
Practical Experience	Have you ever cared for a patient that you believed might have an eating disorder? What did you do?
Cultural Context	How do you think cultural aspects may affect disordered eating in older people? Can you think of any societal factors, or factors that are specific to this age group that may play a part?
Environmental Factors	What aspects of the primary care setting do you think facilitate the identification of eating disorders or disordered eating behaviors in older adults? What barriers make their recognition or management more difficult?
Support Needs	What kind of training, resources or professional support do you think primary care nurses need to improve the identification and management of eating disorders in older adults?

## Data Availability

The full set of anonymized quotations supporting the thematic analysis is not publicly available in order to protect the confidentiality of participants, who belong to relatively small professional communities in which individual identification could be possible. Additional illustrative excerpts are available from the corresponding author upon reasonable request, subject to a confidentiality agreement and for legitimate academic purposes.
